# State Medical Association News

**Published:** 1904-09

**Authors:** 


### State Medical Association News.

Dr. Geo. H. Lee, of Galveston, has been appointed Councilor for Ninth Medical District, vice Decherd, resigned.

Dr. C. E. Cantrell, of Greenville, medical councilor 14th District, has been appointed delegate to the International Congress on Tuberculosis at St. Louis, to meet October 3, 4, 5.

Rr. H. B. Decherd, of Galveston, Councilor for the 9th District, will be absent from the State for a year or more. He has resigned the position, and the appointment of his successor will be announced next month.

The Transactions.—Austin Meeting State Medical Association of Texas, (April, 1904). The big contract, 2550 copies of nearly 700 pages each (illustrated) was awarded to Von Boeckmann-Jones Company, Austin, on competitive bids, in and out of Texas, and they are now, and for some weeks have been, at work on it. The volume is to be uniform with those of the past, handsome brown ribbed cloth and gilt, on excellent paper, and in clean, new sharp type. Members will receive it about November 1st. This house has published the State Association's Transactions many years. They have a splendid equipment and every facility for the work, not the least amongst which is a corps of trained and experienced men and an expert proofreader. They have printed the "Red Back," also, going on 20 years. It speaks for itself. It looks as if no other house in or out of Texas can compete with them. We will get a splendid volume, as usual.

